# Relationship between day 1 and day 2 Vancomycin area under the curve values and emergence of heterogeneous Vancomycin-intermediate *Staphylococcus aureus* (hVISA) by Etest® macromethod among patients with MRSA bloodstream infections: a pilot study

**DOI:** 10.1186/s12879-017-2609-0

**Published:** 2017-08-02

**Authors:** Dmitriy M. Martirosov, Monique R. Bidell, Manjunath P. Pai, Marc H. Scheetz, Susan L. Rosenkranz, Corey Faragon, M. Malik, R. E. Mendes, R. N. Jones, Louise-Anne McNutt, Thomas P. Lodise

**Affiliations:** 1 Albany College of Pharmacy and Health Sciences, Albany, NY 12208-3492 USA; 2 Midwestern University Chicago College of Pharmacy, Downers Grove, IL USA; 3 Northwestern Medicine, Chicago, IL USA; 4 Harvard School of Public Health, Boston, MA USA; 5 JMI Laboratories, North Liberty, IA USA; 6 University at Albany, State University of New York, Albany, NY USA

**Keywords:** Vancomycin, Heteroresistance, *Staphylococcus aureus*, MRSA

## Abstract

**Background:**

In vitro data suggests that suboptimal initial vancomycin exposure may select for heterogeneous vancomycin-intermediate *Staphylococcus aureus* (hVISA) infections. However, no clinical studies have evaluated the relationship between initial vancomycin exposure and emergence of hVISA. This pilot study seeks to assess the relationship between day 1 and day 2 vancomycin area under the curve (AUC) and emergence of hVISA bloodstream infections (BSIs) by Etest® macromethod among patients with a non-hVISA BSI at baseline.

**Methods:**

This was a retrospective cohort study of patients with methicillin-resistant *Staphylococcus aureus* (MRSA) BSIs at Albany Medical Center Hospital (AMCH) between January 2005 and June 2009. The vancomycin AUC exposure variables on day 1 (AUC_0-24h_) and day 2 (AUC_24-48h_) were estimated using the maximal a posteriori probability (MAP) procedure in ADAPT 5.

**Results:**

There were 238 unique episodes of MRSA BSIs during the study period, 119 of which met inclusion criteria. Overall, hVISA emerged in 7/119 (5.9%) of patients. All 7 cases of hVISA involved patients who did not achieve area under the curve over broth microdilution minimum inhibitory concentration (AUC_0-24h_/MIC_BMD_) ratio of 521 or an AUC_24-48h_/MIC_BMD_ ratio of 650. No associations between other day 1 and day 2 AUC variables and emergence of hVISA were noted.

**Conclusions:**

Although more data are needed to draw definitive conclusions, these findings suggest that hVISA emergence among patients with non-hVISA MRSA BSIs at baseline may be partially explained by suboptimal exposure to vancomycin in the first 1 to 2 days of therapy. At a minimum, these findings support further study of the relationship between initial vancomycin exposure and hVISA emergence among patients with MRSA BSIs in a well-powered, multi-center, prospective trial.

## Background

Vancomycin is often employed as a first-line treatment of serious infections due to methicillin-resistant *Staphylococcus aureus* (MRSA) [1]. While susceptibility to vancomycin among MRSA remains near 100%, there are growing report of vancomycin-susceptible strains that exhibit a “heteroresistant” phenotype. Heterogeneous vancomycin-intermediate methicillin-resistant *S. aureus* (hVISA) is defined by the presence of an intermediate-resistant subpopulation at a frequency of 10^−5^–10^−6^ CFU in a susceptible isolate with an MIC_BMD_ ≤ 2 mg/L [2]. Laboratory studies suggest that suboptimal vancomycin exposure early in the course of therapy may select for hVISA during or shortly after completion of therapy [3]. This in vitro finding is concerning as data suggest that patients with hVISA infections are less responsive to vancomycin [4]. Despite this potential association between suboptimal exposure to vancomycin and hVISA emergence, clinical studies evaluating the relationship between initial vancomycin exposure and emergence of hVISA are largely non-existent. This pilot study seeks to assess the relationship between day 1 and day 2 vancomycin area under the curve (AUC) and emergence of hVISA bloodstream infections (BSIs) by Etest® macromethod among patients with a non-hVISA BSI at baseline. Bayesian techniques were used to estimate the vancomycin concentration-time profile for each patient and the relationships between area under the curve (AUC), AUC/MIC, minimum concentration (Cmin), and AUC/MIC and hVISA emergence were examined. The Bayesian approach used to estimate exposure profiles in this study has recently been validated as a method to estimate vancomycin exposure values with low bias and high precision in situations where trough-only PK data are available.

## Methods

This was a retrospective cohort study of patients with MRSA BSIs at Albany Medical Center Hospital (AMCH) between January 2005 and June 2009 performed in accordance with the AMCH Institutional Review Board. The study was approved by expedited review by the Institutional Review Board of AMCH and a HIPAA waiver was obtained. Patients were included if they met the following criteria: (1) age ≥ 18 years; (2) absolute neutrophil count ≥1000 cells/mm^3^; (3) not receiving renal replacement therapy; (4) MRSA blood culture met the Centers for Disease Control and Prevention (CDC) criteria for BSI [5]; (5) index and at least 1 subsequent MRSA bloodstream isolates were available for phenotypic characterization; (6) index MRSA isolate was negative for hVISA by Etest® macromethod [6]; (7) received vancomycin within 48 h of index blood culture; (8) received at least 2 days of vancomycin; and (9) had at least one vancomycin level collected within 5 days of starting treatment.

The study methods are comparable to our recently published study [7]. Isolates were identified as *S. aureus* according to standard methods. Initial susceptibility testing for oxacillin resistance was performed according to CLSI guidelines [8]. Isolates were stored in trypticase soy broth with 20% glycerol at −70 °C. All MRSA bloodstream isolates obtained from the patient were shipped to JMI Laboratories (North Liberty, IA) for hVISA screening and determination of MIC through broth microdilution method (BMD) and Etest® strips (lot# BJ0469) according to the manufacturer’s instructions (bioMérieux, Marcy l’Etoile, France). BMD and Etest® MICs were performed for MRSA using *S. aureus* ATCC 29213 and *Enterococcus faecalis* ATCC 29212 as quality control strains. Screening for hVISA was performed by Etest® macromethod using Mu 3 as a control strain as described by Wootton et al. [6].

All inpatient antibiotic treatment and vancomycin concentration data during the study period were collected. The vancomycin area under the curve exposure variables on day 1 (AUC_0-24h_) and day 2 (AUC_24-48h_) were estimated using the maximal a posteriori probability (MAP) procedure in ADAPT 5 as previously described [7, 9, 10]. This method has been validated to estimate AUC values with low bias and high precision utilizing trough-only sampling [10]. With the Bayesian posterior PK information for a given individual, ADAPT 5 was also used to estimate Cmin_24h_ and Cmin_48h_. The primary vancomycin exposure variables considered in the analyses included: (1) AUC_0-24h_; (2) AUC_0-24h_/MIC_BMD_; (3) AUC_0-24h_/MIC_Etest_; (4) AUC_24-48h_; (5) AUC_24-48h_/MIC_BMD_; (6) AUC_24-48h_/MIC_Etest_; (7) C_min24h_; and (8) Cmin_48h_. We assessed both AUC_0-24h_ and AUC_24-48h_, as AUC_0-24h_ reflects initial exposure conditions and is consistent with the exposure profile evaluated in animal model studies [11, 12], while AUC_24-48h_ represents a near steady-state exposure profile associated with a maintenance vancomycin regimen.

The primary outcome of the study was emergence of hVISA, defined as the documentation of a hVISA bloodstream isolate after 24 h of vancomycin treatment and up to 60 days after completion of therapy among patients with a non-hVISA BSI at baseline. Classification and Regression Tree (CART) analysis was used to identify vancomycin day 1 and day 2 AUC exposure variables associated with an increased probability of hVISA emergence. Bivariate comparisons were assessed using Fisher’s exact test, Student’s t test, and Mann-Whitney *U* test. Poisson regression with robust variance estimates analyses were performed to identify variables independently associated with hVISA emergence. Each CART-derived vancomycin AUC exposure variable was evaluated in a separate regression analysis. All baseline variables associated with emergence of hVISA at a *P*-value <0.2 were considered at model entry and a stepwise approach was employed to identify independent variables. Calculations were performed using SAS version 9.3 (Cary, North Carolina) and CART software (Salford Systems, San Diego, California).

## Results

There were 238 unique episodes of MRSA BSIs during the study period, 119 of which met inclusion criteria. Emergence of hVISA by Etest® macromethod was observed in 7 (5.9%) patients. Nearly all hVISA isolates emerged during vancomycin treatment and 4 of 7 appeared within 15 days of the index MRSA blood culture (Table 1). One case of hVISA emerged after therapy discontinuation. There was a documented intervention in the medical record related to source control in >95% of cases when warranted. In the CART analysis, patients failing to achieve an AUC_0-24h_/MIC_BMD_ ratio of 521 and an AUC_24-48h_/MIC_BMD_ ratio of 650 had increased incidences of hVISA emergence. Among the 61 patients who did not achieve an AUC_0-24h_/MIC_BMD_ ratio of 521 or an AUC_24-48h_/MIC_BMD_ ratio of 650, 11.5% had emergence of hVISA. All 7 cases of hVISA emerged in patients who did not achieve AUC_0-24h_/MIC_BMD_ ratio of 521 or an AUC_24-48h_/MIC_BMD_ ratio of 650; variables were not completely mutually exclusive (Table 1). No associations between other day 1 and day 2 AUC variables and emergence of hVISA were noted (data not shown). No association between Cmin_24h_ and Cmin_48h_ was noted. Among patients with hVISA, the mean (SD) Cmin_24h_ and Cmin_48h_ were 7.3 (3.4) and 13.4 (5.1), respectively. Similar mean (SD) Cmin_24h_ and Cmin_48h_ were observed among non-hVISA patients (8.7 (4.8) and 11.3 (6.0), respectively).

**Table 1:** Clinical characteristics of patients in whom hVISA population was detected

	Patient Number						
	1	2	3	4	5	6	7
AUC0_0-24h_/MIC_BMD_ ratio	420.8	226.4	358.6	519.5	251.9	302.6	759.6
AUC_24-48h_/MIC_BMD_ ratio	506.6	600.9	505.6	806.6	534.7	442.1	545.3
Creatinine clearance (mL/min)	34.2	22.8	34.5	22.1	65.7	25.3	8.4
Days from index MRSA blood culture to emergence of hVISA	62	2	2	14	15	62	23
Days from start of vancomycin to emergence of hVISA	62	2	1	14	13	61	22
hVISA emergence on treatment	Y	Y	Y	Y	Y	N	Y

Abbreviations: *hVISA* heterogeneous vancomycin-intermediate *Staphylococcus aureus*, *AUC*_*0-24h*_ */MIC*_*BMD*_ area under the curve from 0 to 24 h over broth microdilution minimum inhibitory concentration, *AUC*_*24-48h*_ */MIC*_*BMD,*_ area under the curve from 24 to 48 h over broth microdilution minimum inhibitory concentration, *Y* yes, *N*, no

Comparisons between baseline characteristics and emergence of hVISA and day 1 and day 2 AUC/MIC _BMD_ ratio groups and hVISA emergence are shown in Table 2. Compared to their non-hVISA counterparts, hVISA patients were entirely male, had lower baseline mean renal function, and were more likely to have infective endocarditis and receiving immunosuppressive therapy. Relative to patients with an AUC_0-24h_/MIC_BMD_ ratio < 521, patients with an AUC_0-24h_/MIC_BMD_ ratio of ≥521 were significantly more likely to be older and have heart failure, but less likely to have a MIC_BMD_ ≥ 1 mg/L. Patients with an AUC_24-48h_/MIC_BMD_ ratio ≥ 650 were more likely to have recent surgery in the previous 30 days and less likely to have MIC_BMD_ ≥ 1 mg/L relative to those with an AUC_24-48h_/MIC_BMD_ ratio < 650.

**Table 2:** Associations between baseline characteristics and (a) CART-derived AUC_0-24h_/MIC_BMD_ and AUC_24-48h_/MIC_BMD_ groups and (b) emergence of hVISA

	AUC_0-24h_/MIC_BMD_ < 521 (*N* = 52)	AUC_24-48h_/MIC_BMD_ ≥ 521 (*N* = 67)	AUC_24-48h_/MIC_BMD_ < 650 (*N* = 54)	AUC_24-48h_/MIC_BMD_ ≥ 650 (*N* = 65)	Non-hVISA (*N* = 112)	hVISA (*N* = 7)
Age in years, mean (SD)	57.1 (15.6)**	64.5 (14.6)**	58.0 (17.1)*	64.0 (14.4)*	60.8 (15.6)	68.4 (10.9)
Sex-male	34 (65.4)	49 (73.1)	39 (72.2)	44 (67.7)	76 (67.9)1*	7 (100)*
Weight in kg, mean (SD)	86.5 (28.3)	82.6 (31.7)	84.7 (27.9)	84.4 (33.0)	84.0 (30.7)	89.5 (20.5)
Recent residence in healthcare institution in previous 6 months	30 (57.5)	43 (64.2)	27 (50.0)	40 (61.5)	68 (60.7)	5 (71.4)
Diabetes mellitus	19 (36.5)	33 (49.3)*	21 (38.9)	31 (47.7)	48 (42.9)	4 (57.1)
Heart failure	11 (21.2)	27 (40.3)**	13 (24.1)*	25 (38.5)*	37 (33.0)	1 (2.6)
COPD	12 (23.1)	26 (38.8)*	17 (31.5)	21 (32.3)	35 (31.3)	3 (42.9)
Hepatic dysfunction	1 (1.9)	4 (6.0)	1 (1.9)	4 (6.2)	5 (4.5)	0 (0.0)
Malignancy	19 (36.5)	14 (20.9)*	17 (31.5)	16 (24.6)	30 (26.8)	3 (42.9)
Decubitus ulcers	11 (21.2)	14 (20.9)	12 (22.2)	13 (20.0)	24 (21.4)	1 (14.3)
Immunosuppressants	13 (25.0)	10 (14.9)	12 (22.2)	11 (16.9)	20 (17.9)*	3 (42.9)*
HIV	3 (5.8)	1 (1.5)	3 (5.6)	1 (1.5)	4 (3.6)	0 (0.0)
History of cerebrovascular event	5 (9.6)	8 (11.9)	5 (9.3)	8 (12.3)	13 (11.6)	0 (0.0)
Recent surgery in previous 30 days	21 (40.4)	19 (28.4)*	26 (48.1)**	14 (21.5)**	36 (32.1)	4 (57.1)
Recent antibiotics in previous 30 days	29 (55.8)	36 (53.7)	29 (53.7)	36 (55.4)	62 (55.4)	3 (42.9)
Prior vancomycin in past 30 days	11 (21.2)	9 (13.4)	10 (18.5)	10 (15.4)	20 (17.9)	0 (0.0)
Length of stay in days prior to index culture collection, median (IQR) days	2 (0–14)	1 (0–12)	3 (0–19)	1 (0–13)	3 (0–14)*	0 (0–0)*
Intensive care unit at onset	15 (28.8)	26 (38.8)	18 (33.3)	23 (35.4)	40 (35.7)	1 (14.3)
Baseline CL_CR_ in mL/min, mean (SD)	77.8 (56.8)	71.1 (46.9)	80.1 (58.4)	69.8 (47.0)	76.7 (51.5)**	30.4 (17.8)**
APACHE-II Score, mean (SD)	13.3 (5.6)	14.4 (6.5)	14.2 (6.2)	14.1 (6.0)	13.7 (6.1)*	17.7 (4.5)*
MIC_BMD_ ≥ 1 mg/L	16 (30.8)**	8 (9.0)**	16 (29.6)**	6 (9.2)**	18 (16.1)**	4 (57.1)**
MIC_Etest_ ≥ 1.5 mg/L	35 (67.3)*	35 (52.2)*	35 (64.8)	35 (53.8)	65 (58.0)	5 (71.4)
Source of bloodstream infection						
• Intravenous catheter	25 (48.1)*	24 (35.8)*	23 (42.6)	26 (40.0)	48 (42.9)	1 (14.3)
• Skin and soft tissue	13 (25.0)	14 (20.9)	15 (27.8)	12 (18.5)	23 (20.5)**	4 (57.1)**
• Bone and joint	2 (3.8)	6 (9.0)	2 (3.7)	6 (9.2)	8 (7.1)	0 (0.0)
• Respiratory tract	2 (3.8)	7 (10.4)	2 (3.7)*	7 (10.8)*	9 (8.0)	0 (0.0)
• Intra-abdominal	3 (5.8)	4 (6.0)	4 (7.4)	3 (4.6)	6 (5.4)	1 (14.3)
• Urinary tract	1 (1.9)	1 (1.5)	1 (1.9)	1 (1.5)	2 (1.8)	0 (0.0)
• Infected graft/device	2 (3.8)	5 (7.5)	2 (3.7)	5 (7.7)	5 (5.4)	1 (14.3)
• Unknown	4 (7.7)	6 (9.0)	5 (9.3)	5 (7.7)	10 (8.9)	0 (0.0)
Presence of Infective Endocarditis	9 (17.3)	8 (11.9)	9 (16.7)	8 (12.3)	14 (12.6)*	3 (42.9)*

All data presented as number (percent) unless otherwise indicated

*P-value < 0.2

**P-value < 0.05

Abbreviations: *hVISA* heterogeneous vancomycin-intermediate *Staphylococcus aureus*, *SD* standard deviation, *COPD* chronic obstructive pulmonary disease, *HIV* human immunodeficiency virus *IQR* interquartile range, *CL*_*CR*_ creatinine clearance, *APACHE-II* Acute Physiology and Chronic Health Evaluation II, *MIC*_*BMD*_ broth microdilution minimum inhibitory concentration, *MIC*_*Etest*_ Etest® minimum inhibitory concentration

Results of the Poisson regression analyses are shown in Table 3. When controlling for all baseline variables associated with emergence of hVISA at a *P*-value <0.2, patients with a AUC_0-24h_/MIC_BMD_ ≥ 521 had a lower risk of hVISA emergence (relative risk (RR) = 0.14, 95% confidence interval (CI): 0.03–0.60) in the day 1 model. Presence of infective endocarditis, CL_CR_, and skin and soft tissue source of infection were also found to be independently associated with hVISA emergence. In the day 2 model, AUC_24-48h_/MIC_BMD_ ratio ≥ 650 was found to be associated with lower risk of hVISA emergence but the associated *p*-value was 0.08 (*R* = 0.16, 95% CI: 0.02–1.28). Other variables associated with hVISA emergence in the day 2 model included CL_CR_ and presence of infective endocarditis.

**Table 3:** Variables associated with emergence of hVISA in the multivariate analyses

	Variable	Relative Risk	95% confidence interval	*P-value*
Day 1	AUC_0-24h_/MIC_BMD_ ≥ 521	0.14	0.03–0.60	0.008
	CL_CR_	0.93	0.88–0.98	0.004
	Presence of IE	4.94	1.67–14.68	0.004
	Skin and soft tissue source	4.89	1.43–16.71	0.01
Day 2	AUC_24-48h_/MIC_BMD_ ≥ 650	0.16	0.02–1.28	0.08
	CL_CR_	0.95	0.91–0.98	0.007
	Presence of IE	4.62	1.67–12.77	0.003

Abbreviations: *hVISA* heterogeneous vancomycin-intermediate *Staphylococcus aureus*, *AUC*_*0-24h*_ */MIC*_*BMD*_ area under the curve from 0 to 24 h over broth microdilution minimum inhibitory concentration, *AUC*_*24-48h*_ */MIC*_*BMD*_ area under the curve from 24–48 h over broth microdilution minimum inhibitory concentration, *IE* infective endocarditis

## Discussion

This pilot study evaluated the relationship between vancomycin exposure and emergence of hVISA by Etest® macromethod in patients with non-hVISA MRSA BSIs at baseline. Nearly all 7 cases of hVISA emerged during vancomycin treatment and 4 of 7 appeared within 15 days of the index MRSA blood culture (Table 1). One case of hVISA emerged after therapy discontinuation. Using a validated Bayesian technique to estimate each patient’s AUC_0-24h_ and AUC_24-48h_ values with limited PK sampling [10], we found that failure to achieve an AUC_0-24h_/MIC_BMD_ ratio of 521 and an AUC_24-48h_/MIC_BMD_ ratio of 650 was associated with an increased risk of hVISA emergence. Of the 7 patients for which hVISA emerged, 6 occurred in patients with AUC_0-24h_/MIC_BMD_ ratio less than 521 and AUC_24-48h_/MIC_BMD_ ratio less than 650. Interestingly, all 7 cases of hVISA emerged in patients who did not achieve AUC_0-24h_/MIC_BMD_ ratio of 521 or an AUC_24-48h_/MIC_BMD_ ratio of 650 (Table 1), suggesting the dual importance of both day 1 and day 2 vancomycin AUC exposures and cumulative vancomycin exposure over the first 2 days of therapy. Presence of infective endocarditis was also found to be associated with an increased risk of hVISA emergence in the multivariate analyses. This is consistent with a meta-analysis from van Hal and Paterson [13] which described an association between high-inoculum infections (e.g. infective endocarditis) and hVISA. Future studies should aim to better elucidate these findings, including the possibility that an alternative exposure threshold may exist for these patients. The observed associations between decreased baseline creatinine clearance and hVISA infection in the multivariate analyses are also not surprising given the number of reports of resistance emergence in this population throughout the literature [14].

Several things should be considered when interpreting these results. This study is subject to limitations inherent to the study design, namely confounding and selection bias. However, the comparable distribution of baseline characteristics between the CART-derived AUC_24-48h_/MIC_BMD_ groups and persistence of findings in the multivariate analysis suggest that confounding is unlikely to contribute to the exposure-response observed findings. Results may not be applicable to neutropenic and dialysis patients as these patients were excluded from this study; AMCH does not have an appreciable neutropenic population, and exposure variables are numerous and difficult to characterize for hemodialysis-dependent patients (e.g. sporadic dosing, inconsistent removal volumes due to variable durations and filters). Both of these excluded populations are frequently identified at risk for resistance emergence [14] and the incidence of hVISA emergence may be higher in these populations than that observed in our study population. Two study patients had emergence of hVISA within 2 days of vancomycin therapy. It is unclear if suboptimal exposure to vancomycin selected for these isolates or if they were present in a sufficient density for detection at baseline. Clear delineation between exposure accrual and subsequent resistance emergence should be an important consideration in future studies as well.

Our CART-derived AUC_h_/MIC_BMD_ ratio breakpoints for defining hVISA emergence can only be considered a rough estimation due to the small sample size. While vancomycin dose and concentration collections used to generate estimates of vancomycin PK parameters for each individual patient occurred within the first 5 days of therapy, most were obtained on days 1–3. Limiting collection to days 1 and 2 to estimate AUC_0-24h_ and AUC_24-48h_ would have been preferred, but this would have severely restricted our already small study sample. For this reason, we employed a vancomycin population PK model as the Bayesian prior that included creatinine clearance as a covariate to account for potential changes in renal function during the first few days of therapy. As previously described, use of a vancomycin population PK model including creatinine clearance ensured a good fit of the model to the observed vancomycin concentrations collected over the first few days of therapy relative to predicted concentrations in our study population [7]. Future studies should consider intensive PK collections on the day the exposure profile is estimated.

Lastly, hVISA phenotypes were not confirmed by population analysis profile (PAP)-area under the curve (AUC). However, the macrodilution Etest® used in the study has been shown to be a viable screening method with a specificity ranging from 89–98% with few false positives [6]. The presence of hVISA by macrodilution Etest® has also shown to be predictive of patient outcomes across of a number of studies, further highlighting the importance of findings from our pilot investigation and need for further study [13]. Finally, future hypothesis analyzing studies should include DNA fingerprinting of the first isolate and subsequent isolates to ensure that isolates are the same.

## Conclusion

In conclusion, the findings from this pilot suggest that in patients with non-hVISA MRSA BSIs at baseline, failure to achieve an AUC_0-24h_/MIC_BMD_ ratio of 521 or an AUC_24-48h_/MIC_BMD_ ratio of 650 is associated with hVISA emergence by macrodilution Etest®. Though we acknowledge that many factors contribute to emergence of hVISA, and that the occurrence of all outcomes are multi-factorial, our pilot findings strongly suggest that day 1 and day 2 vancomycin AUC profiles may be contributory. Definitive conclusions are limited by the retrospective and exploratory nature of the study and its small sample size. However, these data contribute to existing literature in support of AUC monitoring during the first 48 h of vancomycin therapy in patients with MRSA BSIs [7]. Prospective validation in a larger-scale, multi-center clinical trial is necessary to determine the vancomycin pharmacokinetic/pharmacodynamic profile associated with hVISA emergence given its potentially critical importance for defining early optimal vancomycin therapy.

## Abbreviations

AMCH: Albany Medical Center Hospital
AUC: Area under the curve
AUC_0-24h_/MIC_BMD_: Area under the curve from 0–24 h over broth microdilution minimum inhibitory concentration
AUC_24-48h_/MIC_BMD_: Area under the curve from 24–48 h over broth microdilution minimum inhibitory concentration
BSIs: Bloodstream infections
CART: Classification and Regression Tree
hVISA: Heterogeneous vancomycin-intermediate *Staphylococcus aureus*
IE: Infective endocarditis
MAP: Maximal a posteriori
MRSA: Methicillin resistant *Staphylococcus aureus*
